# The Operative Treatment of Meningitis

**Published:** 1926

**Authors:** E. Watson-Williams

**Affiliations:** Clinical Lecturer in Oto-laryngology in the University of Bristol


### THE OPERATIVE TREATMENT OF MENINGITIS.

BY

E. Watson-Williams, M.C., Ch.M., F.R.C.S.E.,

*Clinical Lecturer in Oto-laryngology in the University of Bristol.*

---

Until recently the outlook in suppurative meningitis was hopeless. Where the infection is diffuse the prognosis is still very bad. But we have learnt that if in the earliest stage, when the trouble is still, we hope, circumscribed, we can remove the infective focus, the patient's chances of recovery are by no means poor. This emphasises the importance of deciding, quite at the beginning of any case of meningitis, whether an otorrhœa may be the *fons et origo mali*, and whether operative treatment should be undertaken; for aural disease is known to cause a large proportion, perhaps the majority, of all cases of septic meningitis.

The decision as to operation may have to be taken before it is possible to determine whether the meningitis is a septic one: to wait until a doubtful case is certain is to throw away the best chance of success. Therefore the value of lumbar puncture in settling the question is limited. If the cerebro-spinal fluid shows manifest changes, that may be an additional reason for operating; but obviously we hope, by basing our decision on physical signs, to anticipate this stage.

Where an expert opinion can be obtained it may possibly save us from uselessly operating on a patient with tuberculous meningitis ; in the one case mentioned below where it might have influenced us the report was not immediately available. It must not be forgotten that in these cases lumbar puncture is not devoid of all risk. It is, however, of great value to the surgeon in determining the type and scope of the operation. For the same reason tests of the labyrinth should never be omitted. The following cases serve to illustrate my thesis.

**Case 1.**—Boy, aged 8. On March 3rd he was admitted under Dr. R. C. Clarke complaining of headache ; the illness began 24 hours before with sudden vomiting. He was drowsy, and resisted examination. There was some head retraction, and Kernig's sign was present ; temp. 102°. Next day he was about the same, but noisy and inclined to scream when roused ; vomiting had ceased. The right ear had been running for five years, but there had been no recent pain there, nor was there any trace of redness or œdema ; there was some resentment to firm pressure, but not more than on the opposite (healthy) side. No nystagmus, normal labyrinthine responses. Clinically the only diagnosis that appeared probable was that of "tuberculous meningitis." Lumbar puncture yielded slightly turbid fluid, under some pressure. It contained 30 cells per c.mm., 40 per cent. lymphocytes, 60 per cent. polymorphs, and was otherwise normal. After consultation, in view of the feeling that the ear *might* perhaps be the source of the trouble, it was decided to operate.

A radical mastoid operation was performed the same evening. The antrum was normal, containing a little mucopurulent fluid. The middle ear contained, deep to the malleus and extending down the Eustachian tube, a cholesteatomatous mass with a very thick wall. This had eroded the roof of the tube, exposing the dura. The dura above the ear, and behind on either side of the sigmoid sinus, seemed perfectly normal.

The boy was no better on the 5th, and seemed even more drowsy on the 6th. It was feared that the original tentative diagnosis would be confirmed ; but on March 8th improvement began, and recovery was thenceforward uneventful.

**Case 2.**—Girl, aged 12. In the same week I was asked to see this child by Dr. Nixon. She had just been admitted with a story of headache for five days, beginning with vomiting. She was drowsy and irritable, but could be roused to answer questions. There was some head retraction, and definite slight tenderness over the right mastoid antrum ; the ear had been running for three months. The labyrinth was normal ; temp. 100·6°. Lumbar puncture gave quite clear fluid under normal pressure. At operation late that night chronic middle-ear disease was found, without antral abscess, and nothing to account for symptoms. Next day Dr. Fraser reported that the cerebro-spinal fluid contained 30 cells per c.mm., 75 per cent. lymphocytes, no organisms: "suggestive of tuberculous meningitis." The girl was decidedly better on the third day, but on the sixth she died of tuberculous meningitis (confirmed at autopsy). This case is mentioned because clinically there seemed nothing by which it might have been distinguished from the first, except that there did appear rather better grounds for believing the meningitis to be of aural origin.

**Case 3.**—Woman, aged 55. She had had a running and deaf left ear all her life, and had three times had an aural polypus removed, the last time on January 30th this year. On February 11th she was brought to the Bristol Royal Infirmary as a case of meningitis. She was complaining of severe headache, frontal and temporal, since the last operation, with shivering attacks the previous night. There had been no vomiting, and she said she had not been giddy. I was called to her on the 12th, and found her in bed, able to lie in any position. There was definite head retraction, doubtful Kernig's sign, increased knee jerks, temp. 101·4°. She was slow in answering and somewhat confused, and groaning with the headache. There was no ear-ache, but definite tenderness over the left mastoid antrum, and a mixed spontaneous nystagmus to the right, worse on looking to the right. Dr. Clarke kindly saw her, and considered there was definite meningitis. Tests of the labyrinth failed to provoke any response. Lumbar puncture yielded quite opalescent (not milky) fluid under pressure ; a film was reported "Many pus cells." Just before going to the theatre, being pressed, she admitted that she had been giddy for several days, though not recently ; tending to fall to the left, but had not fallen. Her husband could not recall any sign or complaint of giddiness.

At operation the same afternoon a very large cholesteatomatous mass was found occupying and expanding the antrum and aditus; the facial nerve ran through it, devoid of any bony support. The stapes was absent, and the oval window exuded pus. The whole labyrinth capsule was carious, and was removed with its contents, and the posterior cranial fossa opened through the internal auditory meatus; a good gush of cerebro-spinal fluid was obtained.

She was very restless all night and noisy; next day there was no pain, no vertigo, no nystagmus; cerebro-spinal fluid was draining well, and the temperature was down. On the third day there was some delirium with return of fever and meningeal signs; the opening was curetted, re-establishing the flow of fluid. From that point she has not looked back.

**Case 4.**—Man, aged 37. Left otorrhœa for many years. He had ear-ache last March with an attack that he called "flu"—vomiting, headache and general malaise. On admission to Southmead Hospital three weeks later the temperature was normal, and he seemed fairly well, but the next day Dr. Phillips found him drowsy and confused. The temperature was 103° and rising, and with this the pulse was getting slower; great complaint of frontal headache, but none of giddiness. I was called to him, and found in addition temporal pain but no trace of mastoid tenderness. No spontaneous nystagmus, but fine nystagmus on looking to the right; the labyrinth was "dead" to all tests. Lumbar puncture gave fluid under considerable pressure, but perfectly clear. His wife then said he had been giddy at the beginning of the illness. A radical mastoid operation showed middle-ear disease, with no trace of the ossicles; a carious patch in the inner antral wall led into a cavity among the bony canals, containing pus: I did a double vestibulotomy. By the third day the temperature was normal, and he was free from pain; the subsequent course has been uneventful. No vertigo followed operation. This might be classified as a case of circumscribed, or possibly threatened, meningitis.

The importance of an early decision on the question of operation in such cases as these is too obvious to need further emphasis. The delay of a few hours may well turn the scale from success to failure. The last two cases also serve to remind us that spontaneous vertigo is *not* a prominent symptom of suppurative endolabyrinthitis. Once disease has destroyed the membranous labyrinth there may be no giddiness when at rest, nor preference for one position of the head. Both the patients at first denied being or having been giddy.

### Summary.

In every case of meningitis in a subject of otorrhœa the question of aural operation should be considered at an early stage.

